# Influence of sound levels, secondary school student characteristics, sound types, and audiovisual interactions on the restorative potential of school environment soundscapes

**DOI:** 10.3389/fpsyg.2024.1476553

**Published:** 2025-02-12

**Authors:** Leyi Zheng, Huanzhen Ren, Shan Shu, Haoyue Gao, Junxi Fan

**Affiliations:** College of Landscape Architecture and Art, Northwest A&F University, Yangling, Shanxi, China

**Keywords:** soundscape, acoustic environment, secondary school student, perceived restorativeness soundscape, influencing factor

## Abstract

**Introduction:**

Soundscapes can significantly impact individuals’ physical and mental health. However, the factors influencing the perceived restorativeness of soundscapes among secondary school students remain unclear. This study aims to explore the effects of school environment sound levels, individual characteristics, types of sounds, and audiovisual interactions on the perceived restorativeness soundscape (PRS) of secondary school students.

**Methods:**

The study design includes measurements of sound pressure levels at 36 locations across six secondary schools in the Yangling District, a questionnaire survey involving 500 secondary school students, and analyses using difference, correlation, and structural equation models.

**Results:**

The school environmental sound level of 59 dB(A) serves as a turning point for PRS. Significant personal factors affecting students’ PRS include gender, stress level, attention level, and noise disruption. Additionally, the frequency of natural and artificial sounds generated by student movements showed a positive correlation with PRS. The combination of audiovisual stimuli was found to enhance PRS among students. Furthermore, the primary factors influencing PRS are the appropriateness of the auditory environment and visual landscape evaluation, followed by the frequency of natural sounds.

**Conclusion:**

Therefore, optimizing school soundscapes requires careful consideration of the appropriateness of the auditory environment, as well as the interest, harmony, and attractiveness of the visual surroundings. It is also crucial to enhance the frequency of natural sounds by incorporating greenery and other strategies. The findings of this study provide a theoretical basis for the optimization of secondary school soundscapes.

## 1 Introduction

In recent years, noise pollution has become a significant concern both domestically and internationally (Aletta et al., 2018). Prolonged exposure to noise can lead to negative emotions such as depression and anxiety (Qiu et al., 2022; Wen et al., 2020) and may also contribute to serious health issues, including tinnitus, heart disease, and cognitive disorders (Sliwinska-Kowalska and Zaborowski, 2017; de Paiva Vianna et al., 2015). Researchers have proposed various strategies to address noise problems and improve mental health, including enacting relevant laws and regulations, mapping urban noise (Di et al., 2018), constructing noise barriers (Petrovici et al., 2016), and constructing an air traffic allocation model using heuristic algorithms to reduce the population’s ability to be exposed to noise (Ganic et al., 2018). In this context, the restorative potential of soundscapes has been emphasized by researchers, which offers a way to reflect on relieving people’s psychological stress and increasing healthy emotions. Research has shown that soundscapes play an important role in environmental perception and have restorative potential for people’s physical and mental health (Szkopiecka et al., 2023; Li Z. Z. et al., 2021; Li S. et al., 2021; Li H. et al., 2021). Given the proven restorative benefits of soundscapes, more research is being focused on the mechanisms that influence the restorative properties of soundscapes to provide practical guidelines for soundscape design.

Soundscape restorative perception is a complex process that involves interactions among the individual, sound, and environment. Each factor influences the final perceptual outcome. At the individual level, an individual’s lifestyle, psychological state, and sociodemographic characteristics affect the perception of sound. Research has shown that age and gender are important factors that influence an individual’s perception of soundscape restorativeness. Education, stress levels, and the frequency of visits to the site (Payne, 2008) are also related to this perception. Various sounds elicit different restorative effects. Natural sounds, particularly bird songs and water sounds, can provide additional restorative benefits. Although musical sounds (Smalley et al., 2023; Steele et al., 2021) and ringtones (Shu and Ma, 2020) have demonstrated their capacity to have restorative effects, studies on the restorative effects of humanistic sounds are still insufficient (Szkopiecka et al., 2023). At the environmental level, the surroundings of sound generation play an important role in the perception of the soundscape with visual factors being a significant part of the context. It was found that more restorative benefits can be achieved and the restorative potential of the environment can be enhanced through audiovisual interactions. For example, discovered that sound inputs are more influential than visual inputs in altering the audiovisual representation of the environment. Similarly, Zhao et al. (2018)found that landscapes featuring a combination of bird songs, water flow, and plants exhibit superior restorative qualities when various combinations of photographs and sounds are used. In addition, the sound level of a different environment affects people’s perception of the environment. Gao Jianmin et al. (2023) found a positive correlation between the comfort of the acoustic environment of Yongjosi Temple and the sound level pressure below 59 dB(A). Zhang et al. (2016) found that at sound levels lower than 60 dB(A), there was a correlation between temple sound levels and comfort evaluations, whereas Ji Xianrong and Yaping (2017) found that school sound levels had a weaker effect on subjective ratings.

Although research has validated the restorative benefits of soundscapes, the factors that influence the PRS need to be clarified (Guo Y. X. et al., 2022; Guo X. et al., 2022). Especially most existing studies focus on adults, fewer studies have been conducted on the soundscape restorativeness of minors, particularly in the secondary school student population. The secondary school stage is a critical period for the rapid development of individuals’ physiology, psychology, and cognition. Studies have shown that in recent years, academic stress has posed health risks to students. Emotions such as depression and anxiety are manifested at young ages, highlighting the need to explore restorative environments to alleviate the stress of secondary school students. The school environment plays a crucial role in students’ academic and personal lives, and a positive school environment is essential for the healthy development of secondary school students. Currently, most soundscape studies focus on university campuses. For instance, Huang et al. (2022) examined the environmental characteristics and sound propagation laws of Guangxi University campus, whereas assessed the soundscape of university campuses and used questionnaire data to investigate the relationship between sound levels and soundscape perception. Although some studies have focused on the acoustic environment of secondary schools, they have primarily studied the average noise levels in classrooms or across the entire school environment, and have explored the restorative effects of music on secondary school students (Kraus et al., 2014; Conservatory of Pavia and Carugno, 2022). The existing studies have not scrutinized the restorative factors of the school environment soundscape on secondary school students. Therefore, it is necessary to explore the mechanisms of perceived influence of soundscape restorability applicable to secondary school students to promote the physical and mental health recovery of this group.

Considering secondary school students as the focus of the investigation, this study aims to investigate systematically the characteristics of the sound level in secondary school environments and explore the various factors influencing secondary school students’ perceived restorativeness soundscape (PRS). Using field measurements and questionnaire surveys of the acoustic environments of six secondary school environments in Yangling District, Xianyang City, this study aims to address the following questions:

1. What are the characteristics of the school environment sound level and how do they relate to the PRS?
2. What is the effect of the individual characteristics of secondary school students on PRS?
3. How do different sound types affect the PRS of secondary school students?
4. What is the relationship between visual attributes, auditory attributes, and PRS by students?

## 2 Materials and methods

### 2.1 Study area

**Figure 1:**
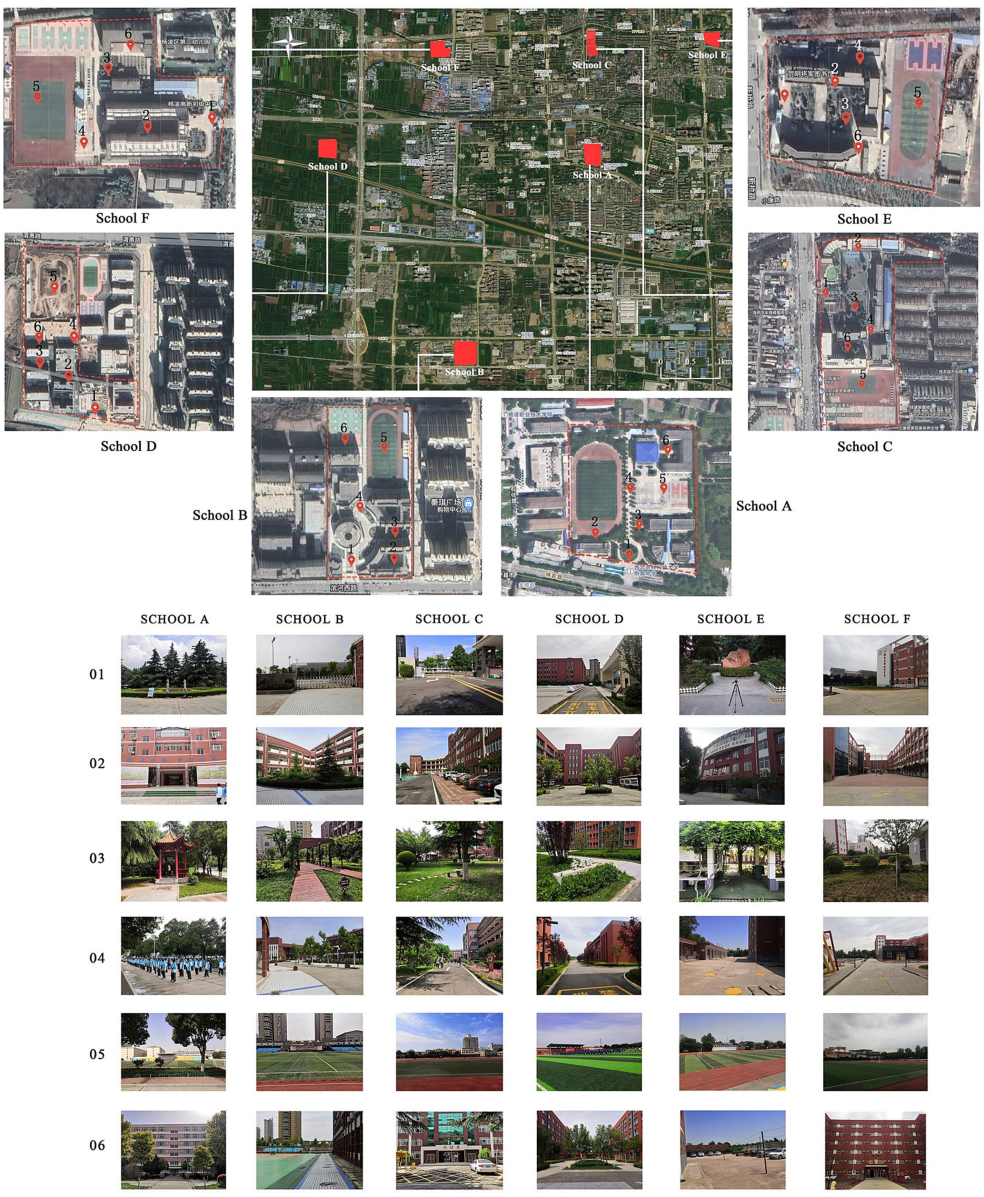
Study area map and measurement point locations (images from OWI interactive maps and author’s field photos; Nos. 1–6 represent the measurement locations for each school).

Yangling District is located in the city of Xianyang in the Province of Shaanxi in the central part of the Guanzhong Plain. It has a long history and covers an area of 132.5685 km^2^. Based on prefield investigations, six representative schools were selected for this study, namely the High-tech High School (School A), Yangling Experimental Secondary School (School B), Yangling High School (School C), Yangling Hengshui Experimental Secondary School (School D), Yangling First Experimental School (School E), and Yangling High-tech Junior High School (School F) (Figure 1). Six measurement locations (Nos. 1–6) were selected for each school based on prefield investigations. No. 1 was located at the entrance of the school, considering the school external and internal environment. No. 2 was near the teaching building, No. 3 was located in the school green area, No. 4 was on the main road of the school, No. 5 was in the playground, and No. 6 was located near the dormitory building. We summarized 18 common sounds, which were divided into three types according to their production modes: natural sounds, artificial sounds, and mechanical sounds. These points essentially covered the characteristics of the acoustic environment of the school (Tables 1, 2).

**Table 1:** Basic information on studied schools.

School	Area (m^2^)	School neighborhood	School environment
School A	70,000	Close proximity to career colleges and neighborhoods.	There are pine trees and shrubs in front of the school, and a pond, gazebo and rockery near the school building.
School B	46,000	Close to wetland Park, gymnasiums and neighborhoods.	There is a fountain at the entrance of the school and an atrium between the school buildings, planted with shrubs and small trees, and equipped with gazebos and arbors.
School C	41,490	Close to stores and kindergarten.	In front of the school building, there is a large centralized green area with deciduous trees, vignettes, and gazebos, and a fountain pool at the side of the entrance.
School D	78,000	Close to a highway, kindergarten, and an elementary school.	This is a new school built in 2019, and the trees have not yet grown and provide poor shade. There are many low shrubs, and the playground and some of the buildings are still under construction.
School E	24,000	Close to institutions and neighborhoods.	There is a centralized green space at the entrance, with planters and vignettes, and a playground adjacent to the school building.
School F	38,667	Close to kindergartens and a middle school.	There is a labor experiment base, lacking trees and shrubs.

**Table 2:** Types of sounds.

Sound classification	Type of sounds
Natural	Birds chirping, rain, running water, leaves blowing in the wind, wind, and insects chirping.
Artificial	The sounds of walking, footsteps, reading, lecturing, playing soccer, playing basketball, playing badminton, and playing table tennis.
Mechanical	The sounds of bell ringing, campus radio, construction noise, recess music, and motorized vehicles.

### 2.2 Questionnaire design

The questionnaire was finalized through modifications, translations, and additions based on previous questionnaires and findings from the preliminary investigations. The reliability and validity of the questionnaire were tested to assess its usefulness before the questionnaires were officially handed out.

The questionnaire was divided into five main parts (Appendix A). The first part contained fundamental data on the secondary school students, including their school, grade, age, and gender.

The second part assessed the state of the secondary school students, including their current stress and attention levels, need for restoration, degree of noise sensitivity, degree of interference by noise, sources of stress, and manners to relieve fatigue. The stress level was based on the DASS-21 medium stress scale with seven questions rated on a four-point Likert scale (0 = disagree to 3 = strongly agree) (Guo Y. X. et al., 2022; Guo X. et al., 2022). The need for restoration, degree of noise sensitivity, and interference (Weinstein, 1978) were consistent with previous study scales. The questions of attention levels and ways to relieve fatigue were designed by our team’s investigators. These factors were assessed according to a five-point Likert scale (1 = strongly disagree to 5 = strongly agree).

The third part focuses on the students’ evaluations of the school visual and auditory environments, employing semantic differential analysis. Evaluations were conducted using a 5-point bipolar rating scale. To accommodate the comprehension abilities and age of secondary school students, we selected vocabulary that is more accessible to them. For visual perception, we identified five pairs of characteristics: interesting–uninteresting, harmonious–chaotic, and attractive–unattractive for visual landscape characteristics (Hong and Jeon, 2013; Jeon et al., 2014), and comfortable–unpleasant and open–closed for spatial characteristics (Grahn and Stigsdotter, 2010). In terms of auditory perception, based on prior studies (Kang and Zhang, 2010; Ma et al., 2018; Jing et al., 2022; Chen, 2021; Yufeng and Xiaofeng, 2020; Lu et al., 2020), we selected 15 pairs of words, including artificial–natural, likable–disgusting, interesting–boring, pleasant–sad, vibrant–listless, comfortable–uncomfortable, concentrated–dispersed, coordinated–disorganized, rich–simple, varied–monotonous, quiet–annoying, friendly–hostile, harmonious–chaotic, safe–dangerous, and weak–strong. These word pairs not only align with international standards for soundscape research but also enhance these standards from psychological, physical, and social perspectives.

The fourth part evaluated the secondary school students’ perceived frequency, loudness, preference, and match of the secondary school students’ perception of sound. These aspects were assessed using a five-point Likert scale. The sound types were summarized from the field research conducted by the prefield investigation investigations. Higher frequency scores indicated that the sound appeared more frequently in the school environment, higher loudness scores indicated that the sound was stronger in the school environment, higher preference scores indicated that the students liked the sound more, and higher matching scores indicated that the sound matched more closely the school environment.

The final part was the perceived restorative rating scale (PRSS), which was partially adapted according to the purpose of the study and the target population. It consisted of five dimensions: fascination (five items), being-away-to (two items), being-away-from (three items), compatibility (two items), and coherence (three items), totaling 15 items. These items were evaluated using a 5-point Likert scale (1 = strongly disagree to 5 = strongly agree). In this case, fascination referred to the phenomenon according to which individuals did not need to focus their attention actively and proactively on a stimulus variable when the environmental elements were interesting. In these cases, attentional resources were not depleted, and fatigue did not occur. Being-away-to and being-away-from referred to mental/cognitive activities that individuals needed to engage in to redirect their focused attention, with the former acting as a pull factor and the latter as a push factor. Compatibility meant that the environment aligned with the individual’s purpose or interests and that a high degree of congruence between the individual’s interests and the environment would reduce the loss of focused attention. Coherence referred to the interconnectedness of elements in the environment, creating a unified whole that enables individuals to explore, observe, and contemplate within it.

### 2.3 Investigation process

Six weekdays were selected in the order of A–F, and each school was surveyed for 1 day from 8:00 a.m. to 10:00 p.m. In total, six investigators were assigned, with each investigator being responsible for one site. First, the investigator identified students willing to fill out the questionnaire at the specific survey location in the school and obtained informed consent from the students. The investigator explained the content, purpose, and precautions of the survey to ensure that the students understood all the questions. Then, the investigator used a Brüel & Kjær 2,250 hand-held noise level analyzer [error ≤ 0.2 dB(A)] to conduct 10-min on-site measurements every 2 h; simultaneously, the students were allowed to wander around the survey site to develop a feel for the surrounding acoustic environment. L_Aeq,10min_ was considered as the overall sound level describing the site soundscape during the assessment period, and the difference between the 10th and 90th percentile levels (L_10-90,10min_) was used to describe the temporal changes in the sound environment. At the end of the sound pressure level measurements, the investigators recorded the L_Aeq,10min_ and L_10-90,10min_ data and collected the questionnaires. The conversations between students and investigators did not influence the sound level measurement because they never occurred in the immediate vicinity of the person holding the sound level meter in his hands during the 10-min period; in addition, these conversations were conducted essentially in low voice by a different investigator. Moreover, during the process of filling out the questionnaire, students who felt uncomfortable could withdraw at any time, in which case the questionnaire was considered invalid. Note that because the survey was conducted by randomly searching for students to fill out the questionnaire within the school, students were not always available to fill out the questionnaire at each sound pressure level measurement, and most of the time, the survey was conducted within 10–12 pm and 14–16 pm, with roughly 12–14 questionnaires being collected at each location (Appendix B).

During the survey period, the outdoor wind speed was less than 5 m/s, the weather was clear and cloudless, and the sound pressure level was ensured to be 1.2 m from the ground and at least 1 m from the surface of the building (Zhang et al., 2016).

Questionnaires were distributed to 500 students; 472 valid questionnaires were returned with a validity rate of 94.4% (School A: 73, School B: 88, School C: 80, School D: 77, School E: 75, and School F: 79). The respondents comprised 224 boys and 248 girls aged 12–18 years (mean age of 15.26 ± 1.53). The reliability and validity tests of the questionnaire showed that Cronbach’s alpha ranged from 0.775 to 0.893 and Kaiser–Meyer–Olkin ranged from 0.697 to 0.908. These results indicate that the questionnaire data were accurate and reliable (Appendix B).

### 2.4 Data analysis

First, descriptive statistics were used to analyze the characteristics of L_Aeq,10min_ and L_10-90,10min_, and Spearman correlation analyses were conducted to explore the relationship between sound levels, L10-90, and the PRS. Second, Mann–Whitney *U*-tests and Spearman correlation analyses were used to analyze the relationship between individual characteristics and PRS. Third, Kruskal–Wallis analysis was used to compare natural, artificial, and mechanical sounds. Spearman correlation analyses investigated the connection between specific sounds and PRS. SPSS (version 26.0, IBM Corporation, Armonk, NY, USA) and Origin (version 2021, OriginLab Corporation, Northampton, Massachusetts, USA) were used for the above data analyses and plotting. Fourth, exploratory factor analysis (EFA) was used to extract the main factor, and confirmatory factor analysis (CFA) was used to verify the factor structure. On this basis, the structural equation model (SEM) was constructed to explore the relationship between audiovisual interactions and PRS. Intermediate effects analysis was used to confirm the mediating role of visual effects in the generation mechanism. The AMOS software program (version 28.0, IBM Corporation, Armonk, NY, USA) was used to analyze confirmatory factor analysis and SEM, and the Visio software program (version 2019, Microsoft Corporation, Redmond, Washington, USA) was used to draw model diagrams. Finally, SPSS was used to construct a regression equation to analyze the key factors affecting PRS.

## 3 Results

### 3.1 Relationship between school environment sound pressure levels and PRS

**Figure 2:**
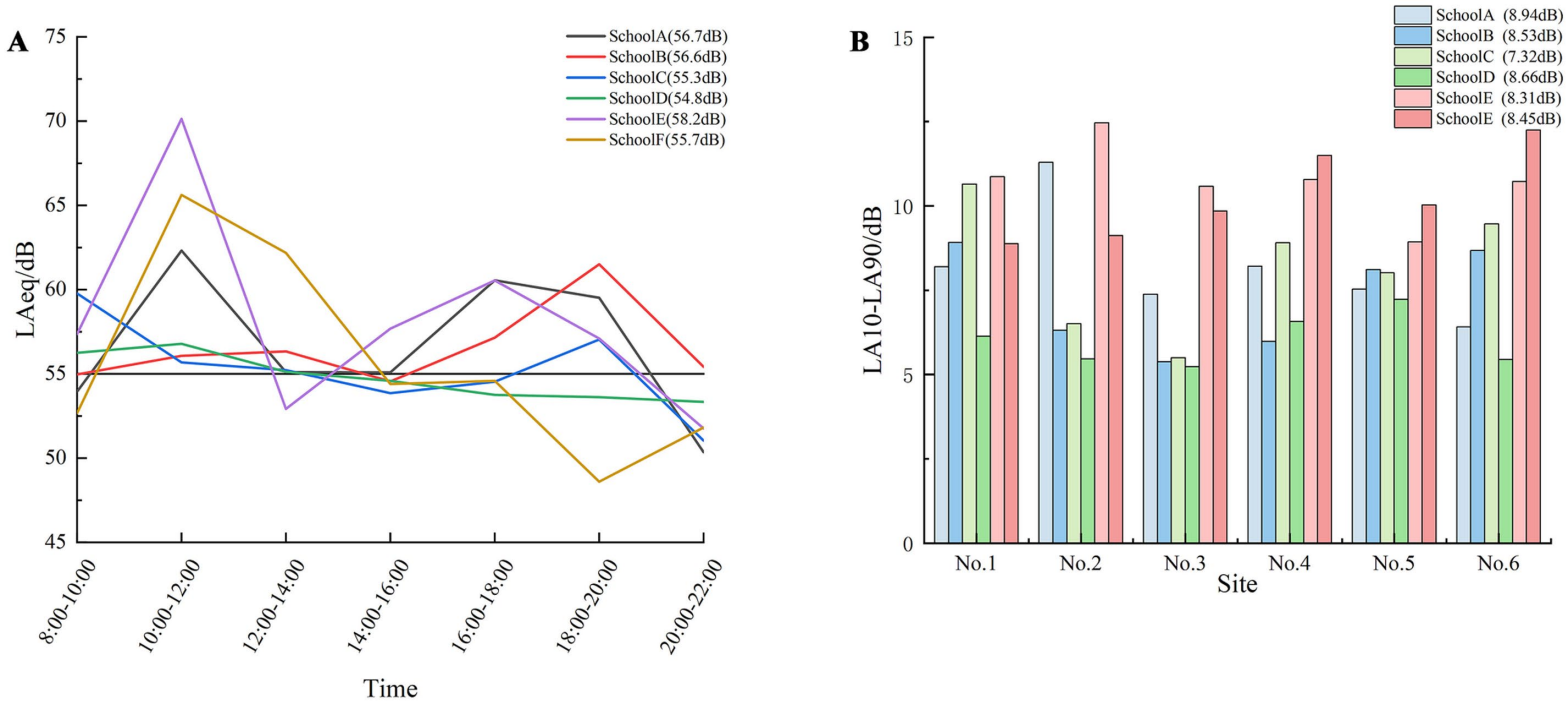
Trends of sound pressure levels and L10-90 in six schools. **(A)** Sound pressure level and **(B)** L10-90.

The results of the Kruskal–Wallis test indicated no significant difference in L_Aeq,10min_ among the six schools (*H* = 6.036, *p* = 0.303). The overall L_Aeq,10min_ ranged from 54.8 to 58.2 dB(A), with an average of 56.2 dB(A). At School E, the sound pressure level between 10:00 and 12:00 was significantly higher than those recorded between 12:00 and 14:00, and between 20:00 and 22:00 (*H* = 16.495, *p* = 0.011). Similarly, at School F, the sound pressure level from 10:00 to 12:00 was significantly higher than that from 18:00 to 20:00 (*H* = 17.666, *p* = 0.007). A significant difference was also observed among the six schools regarding L_10-90.10min_ (*H* = 35.545, *p* < 0.05). The L_10-90,10min_ range was between 7.32 and 8.94 dB(A) [Mean = 8.37 dB(A)]. School C exhibited the smallest L_10-90_ range [Mean = 7.32 dB(A)], indicating the smoothest variation in sound levels. In particular, location No. 3 had the smallest L_10-90,10min_ range [Mean = 7.32 dB(A)], suggesting that the sound level changes in green school environment spaces were the most consistent (Figure 2).

**Figure 3:**
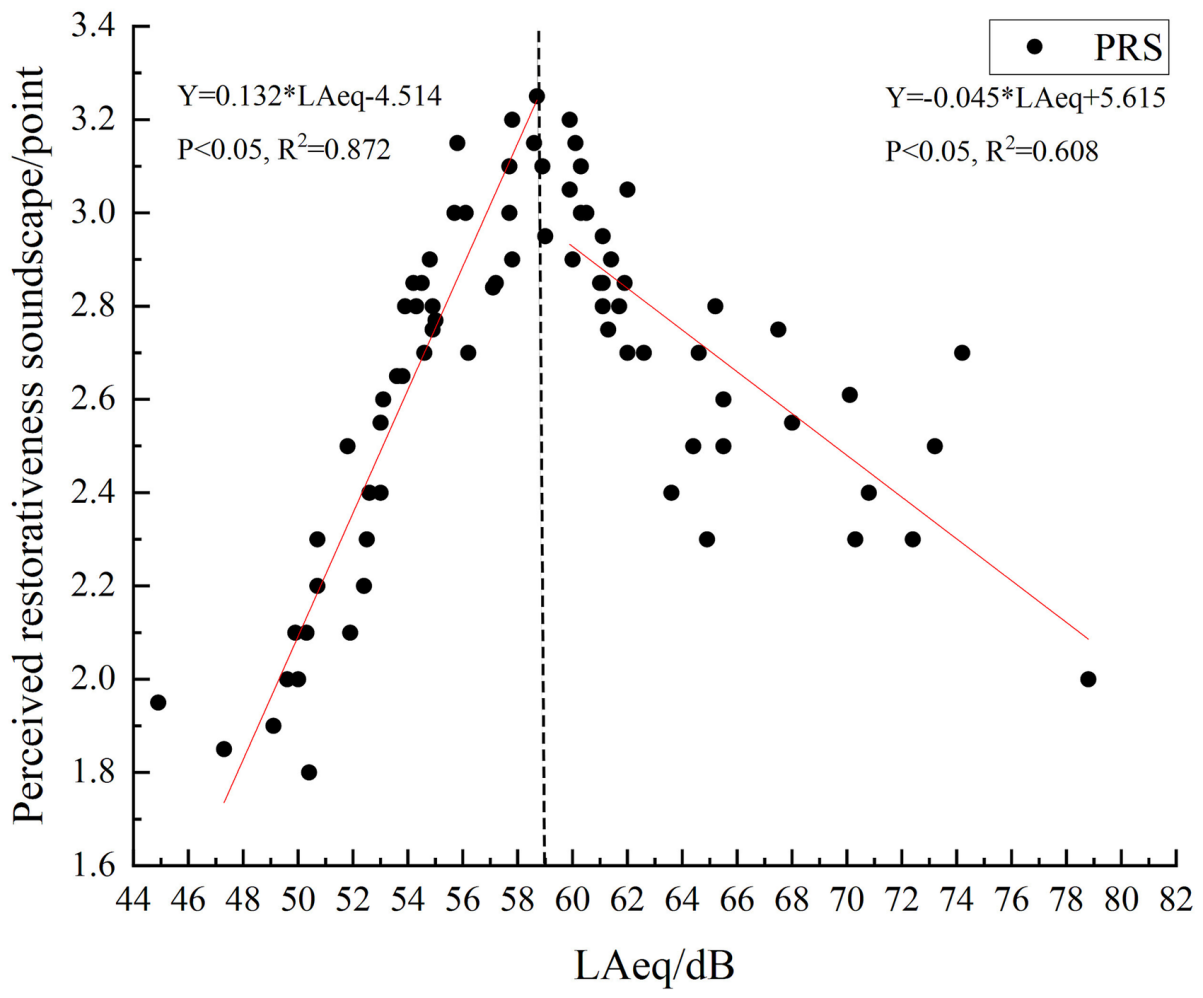
Relationship between sound pressure levels and perceived restorativeness soundscape.

Upon exploring the relationship between the average value of the PRS (Mean = 2.65, standard deviation = 0.36) during the evaluation period and the corresponding sound pressure levels at specific locations and times, a correlation was found between the PRS and sound pressure level (*R* = 0.228, *p* = 0.045). A scatter plot illustrated this relationship, indicating that the PRS first increased and then decreased. Specifically, when the sound pressure level was below 59 dB(A) (*R* = 0.921, *p* < 0.05), the PRS increased with increasing sound pressure levels. However, above 59 dB(A) (*R* = −0.787, *p* < 0.05), PRS decreased as the sound pressure levels increased (Figure 3). No correlation was found between L_10-90_ and the PRS (*p* > 0.05).

### 3.2 Relationship between personal factors and PRS

**Figure 4:**
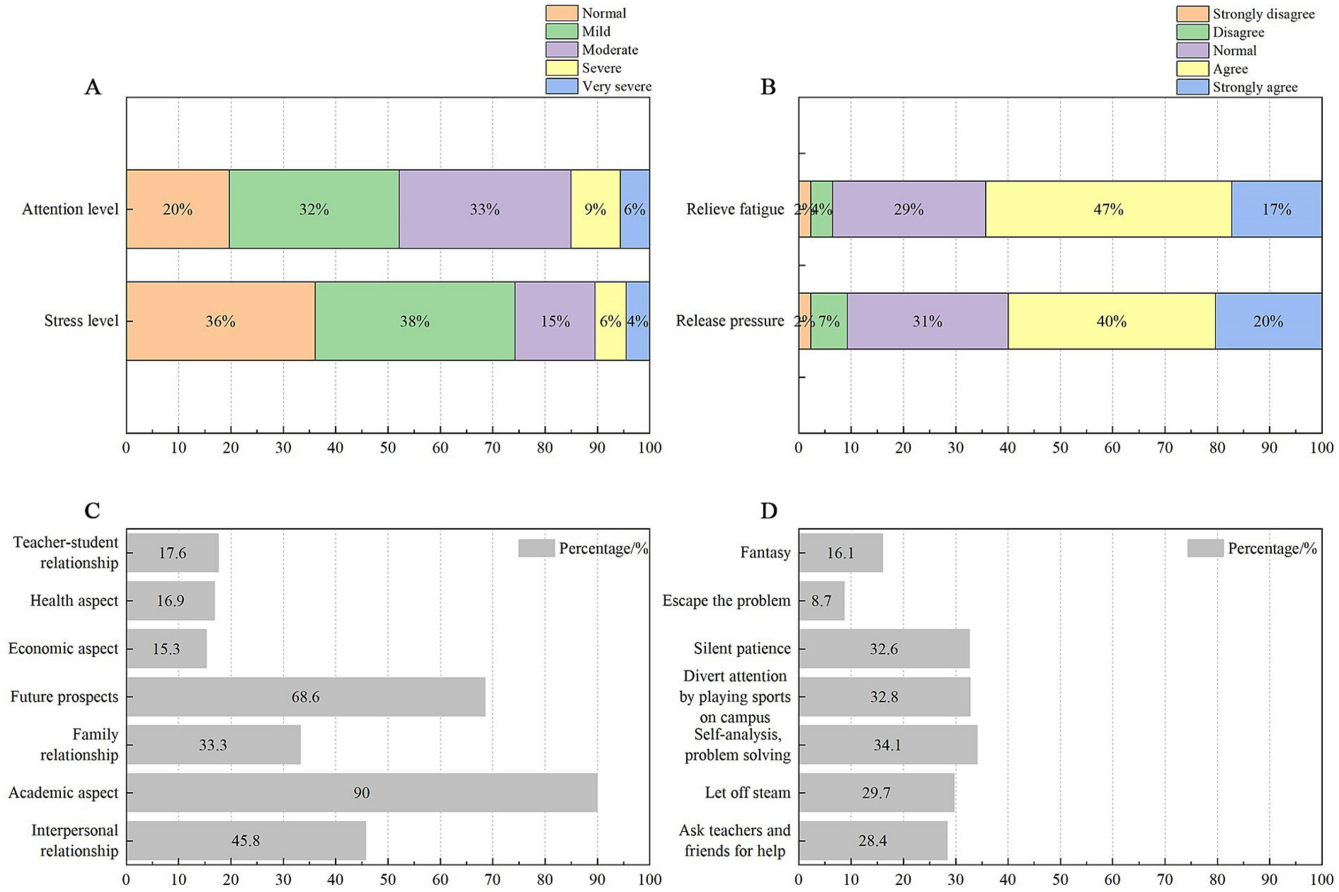
Personal information. **(A)** Attention and stress levels, **(B)** Stress and fatigue relief, **(C)** Sources of stress, and **(D)** Manners to relieve stress and fatigue.

The data indicated that only 20 and 36% of the students reported normal levels of stress and concentration, respectively. The majority of the students experienced stress and fatigue, with 4% feeling extremely stressed and 6% feeling particularly tired. Academic pressure and concerns about future development were identified as the primary sources of this stress. Nearly 60% of the students expressed a need for methods to relieve pressure and fatigue. Most of them indicated a preference for analyzing the sources of their stress and fatigue, making appropriate assessments, confronting problems directly, and responding positively. Others opted for physical activities, such as engaging in sports or walking around school, to divert their attention (Figure 4).

Table 3 presents the results of the correlation and difference tests (gender) between the personal characteristics of secondary school students and PRS. Girls (mean = 2.75, standard deviation = 0.033) achieved higher PRS outcomes than boys (mean = 2.59, standard deviation = 0.041) and rated more favorably the acoustic environment of the school environment (*Z* = −2.883, *p* < 0.01). Stress levels were negatively correlated with PRS (*R* = −0.149, *p* = 0.001), fascination (*R* = −0.102, *p* = 0.026), being-away-from (*R* = −0.150, p = 0.001), compatibility (*R* = −0.177, *p* < 0.01), and coherence (*R* = −0.105, *p* = 0.023). Attention levels also exhibited negative correlations with PRS (*R* = −0.122, *p* = 0.008), compatibility (*R* = −0.091, *p* = 0.049), and coherence (*R* = −0.135, *p* = 0.003). Restorative needs were negatively correlated with being-away-from (*R* = −0.121, *p* = 0.008), compatibility (*R* = −0.138, *p* = 0.003), and coherence (*R* = −0.092, *p* = 0.046). Additionally, noise disturbance showed negative correlations with PRS (*R* = −0.168, *p* < 0.01), being-away-from (*R* = −0.170, *p* < 0.01), compatibility (*R* = −0.215, *p* < 0.01), and coherence (*R* = −0.209, *p* < 0.01). No significant relationship was found between noise sensitivity and PRS.

**Table 3:** Correlation and difference analysis between individual factors and perceived restorative soundscapes (PRS).

Variable	Gender	Age	Stress level	Attention level	Restorative needs	Noise disturbance	Noise sensitivity
PRS	−2.883**	−0.015	−0.149**	−0.122**	−0.087	−0.168**	−0.040
Fascination	−2.413*	−0.034	−0.102*	−0.085	0.009	−0.068	−0.011
Being-away-to	−0.078	−0.014	−0.031	−0.041	−0.036	−0.021	0.070
Being-away-from	−1.821	−0.009	−0.150**	−0.090	−0.121**	−0.170**	−0.042
Compatibili-ty	−1.793	−0.005	−0.177**	−0.091*	−0.138**	−0.215**	−0.075
Coherence	−4.018**	−0.032	−0.105*	−0.135**	−0.092*	−0.209**	−0.061

*p < 0.05, **p < 0.01.

### 3.3 Relationship between different sound sources and PRS

**Figure 5:**
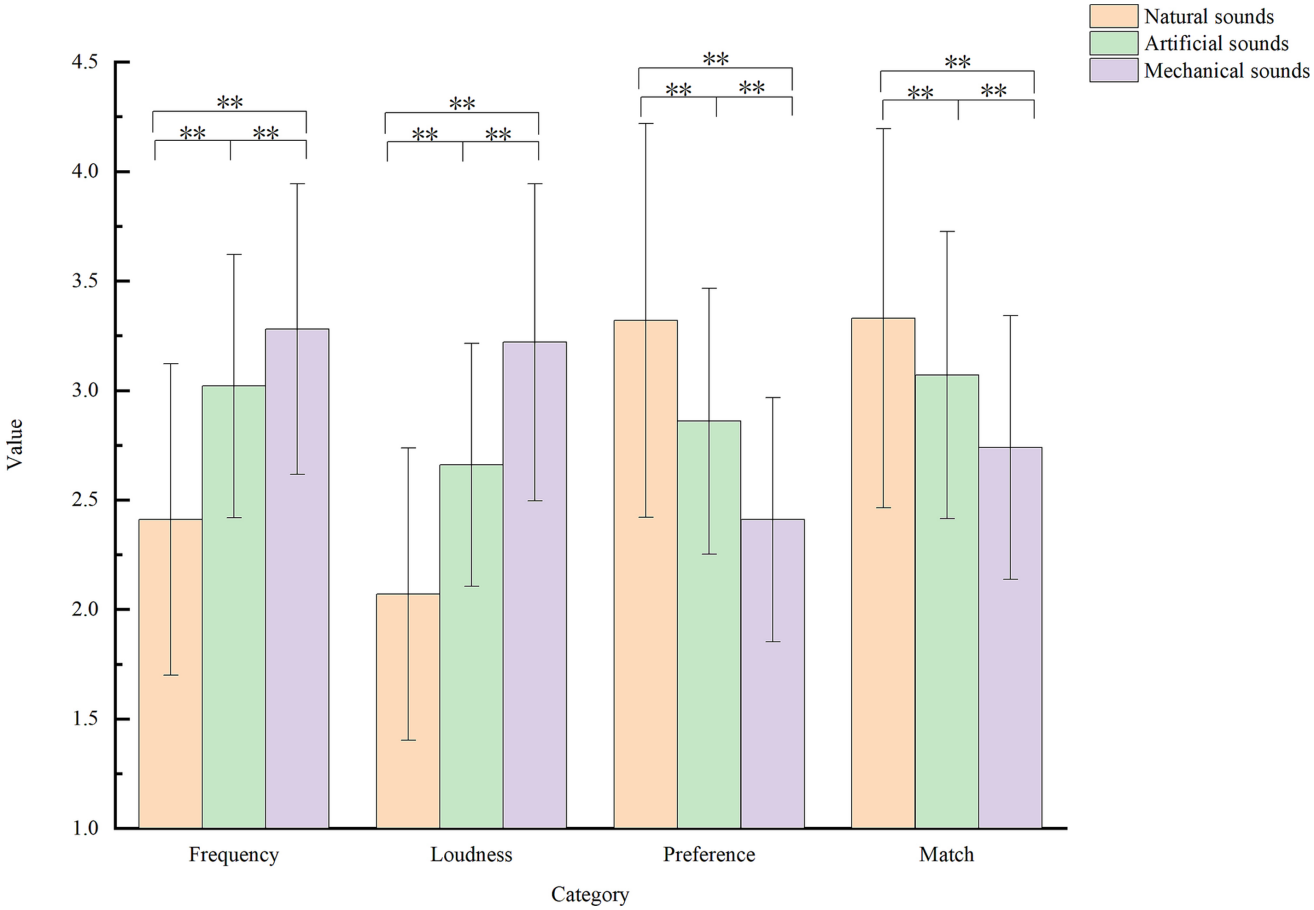
Descriptive statistics for sound source category perception (*p < 0.05,**p < 0.01).

**Figure 6:**
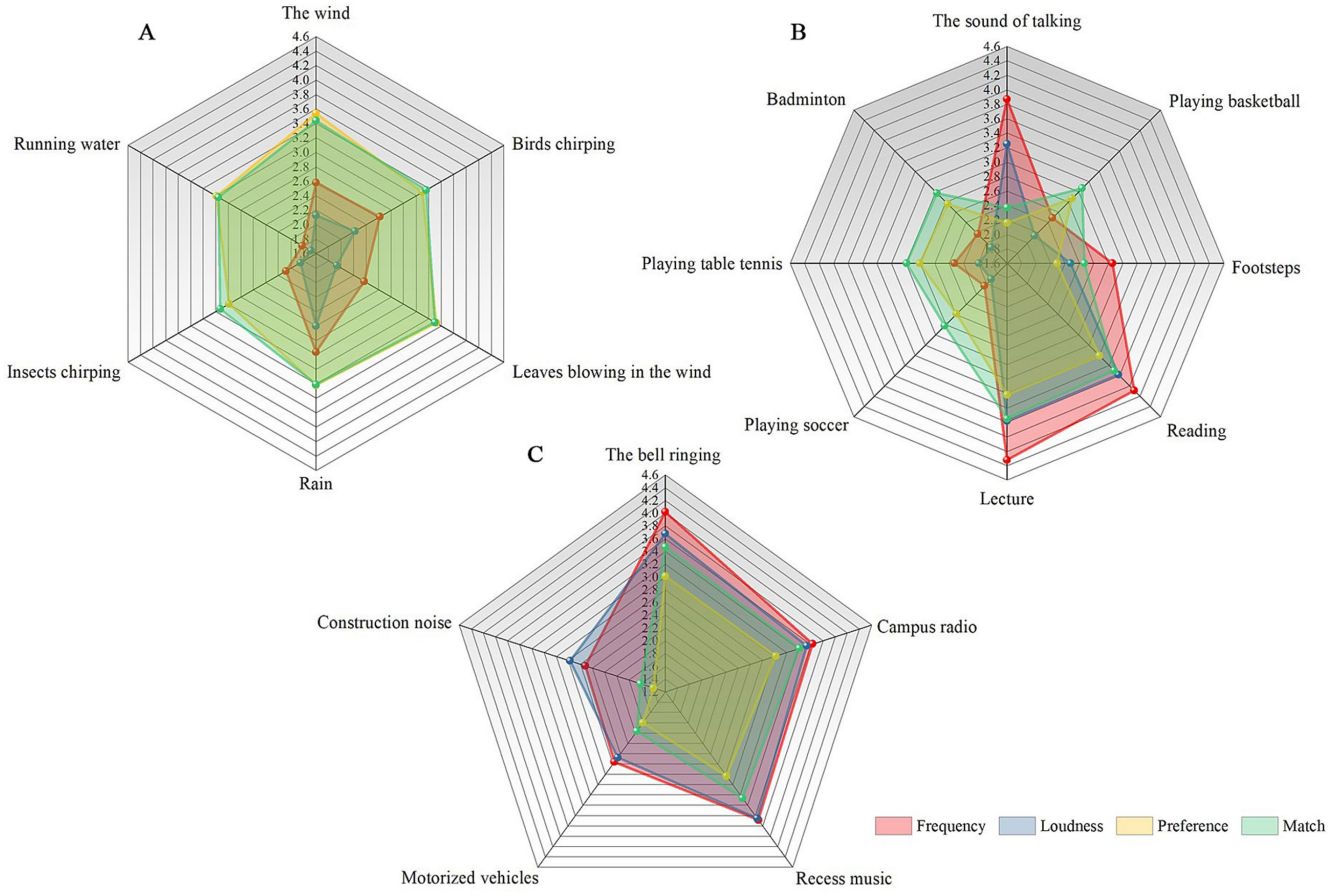
Mean values of frequency, loudness, preference, and match for different sound categories. **(A)** Natural sounds, **(B)** artificial sounds, and **(C)** mechanical sounds.

Natural sounds were the least frequent (2.41 ± 0.711) and loudest (2.07 ± 0.667) in the school environment. However, the students showed a high preference for natural sounds (3.32 ± 0.899) and high-match ratings (3.33 ± 0.865). The opposite was true for mechanical sounds. Artificial sounds were located between the two sound sources (Figure 5). Among the natural sounds, the sound of rain had the highest frequency (2.96) and loudness (2.60). The sound of wind (3.54) and leaves blowing in the wind (3.52) were more preferred, with the sound of leaves blowing in the wind (3.5) having higher degrees of matching. Among the artificial sounds, the lecture sound had the highest frequency (4.32), loudness (3.78), preference (3.42), and match (3.75), followed by the outcomes of the reading sound (4.09, 3.78, 3.41, and 3.70). Among the mechanical sounds, the bell ringing had the highest frequency (4.02) and loudness (3.68), followed by the sound of recess music (3.68 and 3.65). The preference (3.02) for the sound of the campus radio was the highest, and the match for the sound of the bell ringing was the highest (3.47), followed by the sound of campus radio (3.41) (Figure 6).

**Figure 7:**
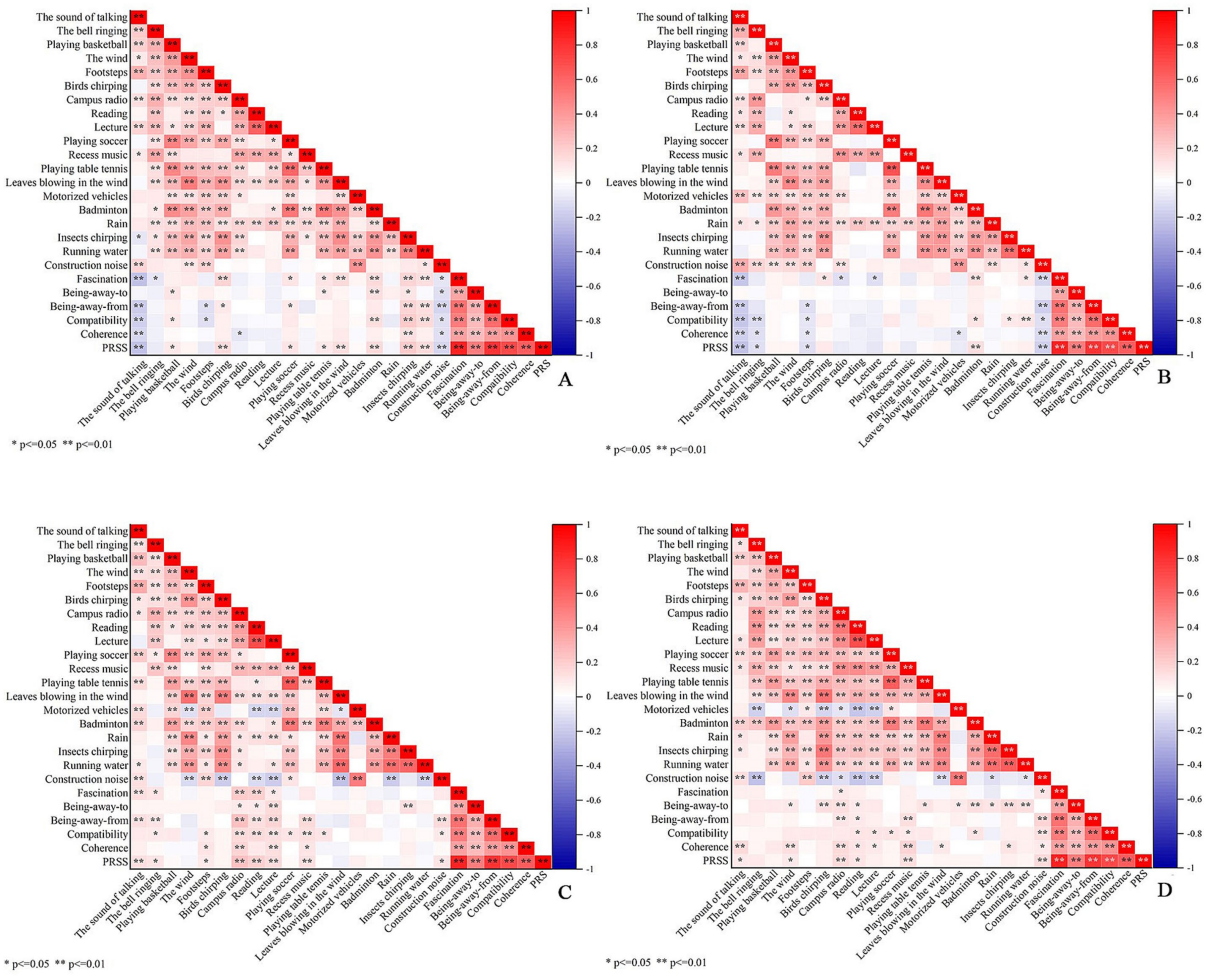
Relationship between different sound categories and PRS. **(A)** Frequency, **(B)** loudness, **(C)** preference, and **(D)** matching.

The correlation results (Figure 7) showed that in the frequency analysis, the sounds of birds chirping, leaves blowing in the wind, insects chirping, running water, badminton, basketball, table tennis, and soccer were positively correlated with the PRS, with larger correlation coefficients for the sounds of insects chirping (*R* = 0.257, *p* < 0.01) and running water (*R* = 0.242, *p* < 0.01). The sound of talking, footsteps, bell ringing, campus radio, and construction noise were negatively correlated with the PRS, with the sound of talking (*R* = −0.233, *p* < 0.01) having the largest correlation coefficient. In the loudness analysis, the sounds of talking, footsteps, bell ringing, and construction noise were negatively correlated with the PRS, with the sound of talking (*R* = −0.233, *p* < 0.01) having the largest correlation coefficient. Conversely, the sound of playing badminton was positively correlated with the PRS (*R* = 0.122, *p* = 0.008). In the preference analysis, the sounds of talking, footsteps, reading, lecturing, bell ringing, campus radio, recess music, and construction noise were positively correlated with the PRS, with the sound of campus radio (*R* = 0.249, p < 0.01) having the largest correlation coefficient. In the match analysis, the sounds of wind, talking, reading, campus radio, recess music, and construction noise were positively correlated with the PRS, with the campus radio (*R* = 0.145, *p* = 0.002) having the largest correlation coefficient.

### 3.4 Relationship between audiovisual interaction and PRS

#### 3.4.1 Validation factor analysis

Based on factorial analysis, visual perception was categorized into two dimensions: spatial evaluation of landscape features and visual landscape evaluation. These factors accounted for 74.67% of the variance. Auditory perception was divided into five dimensions: appropriateness, stability, originality, richness, and harmony. These factors accounted for 68.26% of the variance (Appendix C).

The tests of the visual, auditory, and soundscape restorative perception scales demonstrated acceptable convergent validity and combined reliability [standardized factor loadings ≥0.5, average variance extracted (AVE) ≥ 0.5, and construct reliability ≥0.6]. The standardized correlation coefficients of the dimensions between the two were less than the square root of the AVEs to which the dimensions corresponded, indicating good discriminant validity (Appendix D).

**Figure 8:**
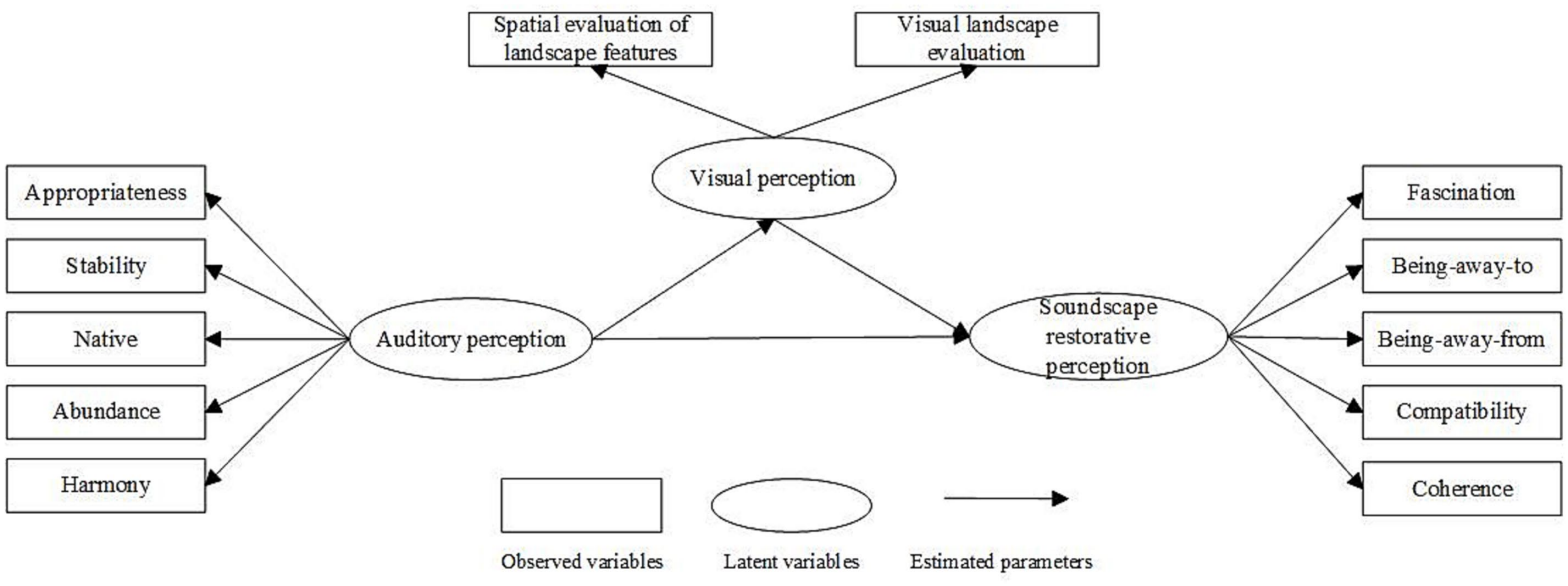
Metamodeling of audiovisual interaction and perceived restorativeness soundscape.

According to previous studies, both visual landscapes and soundscapes have restorative effects. The perception process involves an interaction between the two. Based on this interaction, a meta-model of audiovisual interaction and soundscape restorative perception was constructed (Figure 8), and three specific hypotheses were proposed:

*H1*: Auditory perception has a positive effect on visual perception.

*H2*: Auditory perception has a positive effect on the PRS.

*H3*: Visual perception has a positive effect on the PRS.

#### 3.4.2 Modeling audiovisual interaction and PRS

**Figure 9:**
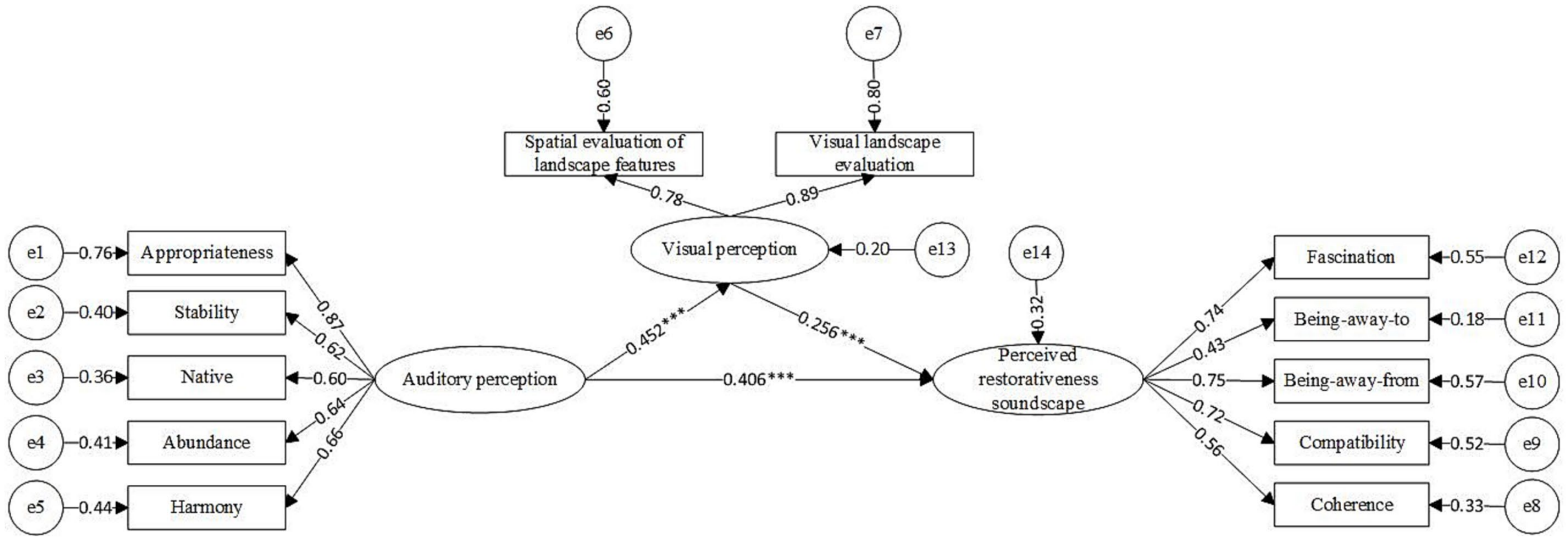
Modeling the effects of audiovisual interactions on perceived restorative soundscapes (*p < 0.05, **p < 0.01, ***p < 0.001).

The model was tested for fitness (Kelly and Walton, 2021) and exhibited a chi-square degree of freedom ratio (CMIN/DF) of 1.917 and root-mean-square of error of approximation (RMSEA) of 0.044, as well as an incremental fit index (IFI = 0.984), Tucker–Lewis index (TLI = 0.979), and comparative fit index (CFI = 0.984) above 0.9, suggesting a good fit. Thus, a structural equation model was derived (Figure 9).

All three hypotheses were tested for significance, and all were positively correlated. Both auditory perception (*β* = 0.406, *p* < 0.001) and visual perception (β = 0.256, *p* < 0.001) had a significant positive impact on the PRS with auditory perception demonstrating the greatest effect. In addition, auditory perception also had a positive effect on visual perception (β = 0.452, *p* < 0.001). Among the auditory perception observation variables, appropriateness was the largest loading factor (0.87), followed by harmony. Among the observed variables of visual perception, visual landscape evaluation (Table 4) was the largest loading factor (0.89).

**Table 4:** Path test analysis of a model of the effect of audiovisual interaction on the perceived restorative soundscapes.

Observed variables	Estimate	SE	CR
Ap — > Vp	0.452^***^	0.085	7.535
Ap — > PRS	0.406^***^	0.044	6.294
Vp — > PRS	0.256^***^	0.029	4.255

*p < 0.05, **p < 0.01, ***p < 0.001. Ap, Auditory perception; Vp, Visual perception.

The validity of the model was analyzed. The *R*^2^ for visual perception was 20.4%, indicating that the model auditory perception could account for 20.4% of the PRS variable variance. Additionally, the *R*^2^ for PRS was 32.5%, indicating that the auditory and visual perception variables together accounted for 32.5% of the variance of PRS.

According to Preacher and Hayes (2008), who proposed bootstrapping repeated sampling (2000 times) to explore the mediating effects of visual perception (Table 5), neither the direct (*p* < 0.05) nor indirect (*p* < 0.05) effect estimates included zero within the 95% confidence interval, which suggests that visual perception played a partially mediating role. Auditory stimuli perceived by the secondary school students directly contributed to their PRS. Conversely, these stimuli contributed indirectly to restoration through specific visual sensory stimuli.

**Table 5:** Mediating effect test of visual perception on the relationship between auditory perception and perceived restorativeness soundscape.

Effect	Path	Estimate	SE	Bias-corrected 95% confidence interval		*p*-value	Effect ratio
				Lower	Upper		
Indirect effect	Ap— > Vp— > PRS	0.080	0.023	0.039	0.132	0.001	13.3%
Direct effect	Ap— > PRS	0.280	0.051	0.187	0.386	0.001	77.8%
Total effect	Ap— > PRS	0.360	0.050	0.265	0.464	0.001	

## 4 Discussion

### 4.1 Effects of sound level on PRS

Because sound pressure levels did not significantly differ among the six schools (*H* = 6.036, *p* = 0.303), we can consider them collectively. The average L_Aeq,10min_ values for most schools were slightly higher than the daytime sound pressure level standard [55 dB(A)], reported by the Acoustic Environment Quality Standard for Class 1 functional areas. Consequently, some noise reduction measures should be implemented, including reasonable control of school construction times and the use of double-glazing windows. Overall, the trend in sound pressure levels is closely related to students’ working hours, with levels between 10:00 and 12:00 significantly higher than at other time points. The time variation in the acoustic environment differs among schools. School C exhibited the smallest time variation in its acoustic environment, resulting in relatively smooth changes; this finding is likely related to its large green spaces, water features, and distance from main roads. In the school environment, the variation in sound levels in green recreation areas was minimal, possibly owing to the surrounding vegetation, which provides shielding and reduces interference.

The subjective evaluation of soundscapes is also closely linked to the average L_Aeq,10min_ value, particularly when sound pressure levels fall below a certain threshold. For instance, the acoustic comfort of open public spaces in European cities corresponds to 57 dB(A), while typical urban green spaces in Chengdu correspond to 77 dB(A) (Shao et al., 2022). In Han nationality Buddhist temples, sound pressure levels were measured at 60 dB(A) (Zhang et al., 2016). Most existing research has focused on establishing the relationship between soundscape comfort and various environments. The PRS, as a subjective evaluation, should correlate with the average L_Aeq,10min_ value. This study constructed the relationship between the physical environment and subjective perception, complementing the research conducted by Guo et al. (2022). The results indicate that at an L_Aeq,10min_ of 59 dB(A), there is a positive correlation between L_Aeq,10min_ and the PRS of the sound environment. Above this threshold, PRS decreases as L_Aeq,10min_ increases.

### 4.2 Influences of personal factors on PRS

This study, which focused on students (ages: 12–18 years), refines previous research (Guo et al., 2022) by examining the perceived restorative soundscape of this particular group. Currently, secondary school students primarily experience pressure related to academic performance and future educational opportunities. In their daily studies, many students encounter stress and fatigue. However, they often employ proactive coping strategies. Demographic factors indicate that gender differences have been confirmed in previous research, both in adults (Liu Jiang et al., 2022) and in children (Shu, 2023). Girls reported a more favorable PRS of the school’s acoustic environment and thought that the acoustic environment of the school was appealing and consistent. Interestingly, the PRS of students of different ages did not exhibit correlations; this finding contradicts earlier studies (Erfanian et al., 2021; Shu, 2023). For instance, some research suggested that individual evaluations of pleasantness and restorative needs of soundscape tended to increase with age, with pleasantness being a crucial factor in how people perceive restorative soundscapes (Herranz-Pascual et al., 2019). This discrepancy may arise because most secondary schools comprise both junior and senior high school students, who often share similar perceptions of the school environment owing to overlapping school activities and familiarity with the schools.

The results regarding the psychological state of secondary school students indicated that students with higher levels of stress and attention tend to have lower PRS, which contrasts with some research findings. This may be because most students’ stress comes from their academic work (Deb et al., 2015). The greater the academic pressure, the more time and energy students invest in their studies, which leads to increased anxiety and suppressed emotions. Consequently, students spend less time experiencing the school environment, which contributes to lower evaluations of the school environment PRS. Therefore, secondary schools should respond to the national “Double reduction” policy and, to some extent, reduce the students’ academic burden and balance the need for good learning and rest. This approach will enable students to relax within the school environment and promote their healthy development. Additionally, a negative correlation existed between the level of noise interference and PRS. This finding reflects the fact that the surrounding acoustic environment affects students’ learning experiences. The study indicates that as noise interference increases, students’ PRS decreases. This places higher demands on the existing sound environment to reduce external noise interference and add a restorative soundscape.

### 4.3 Effects of different soundscapes on PRS

The results of the study revealed that the frequency and loudness of natural sounds on secondary school environments were low. However, the students expressed a strong preference for natural sounds, considering them more suitable for the school environment. This finding aligns with previous studies (Luo et al., 2023; Ma et al., 2021; Shu and Ma, 2020; Krzywicka and Byrka, 2017) indicating that people generally favor natural sounds. Among these, the sound of running water has the lowest frequency and loudness. Although many schools feature pools and fountains, these amenities are typically only accessible on special occasions. Consequently, there is a need to increase the number of operational fountains or incorporate running water features more frequently. The sounds of running water and fountains have been shown to provide significant restorative and shelter effects (Shu and Ma, 2020). Students preferred the sound of leaves blowing in the wind and thought that this sound was more compatible with the school environment. Therefore, it is essential to increase the presence of plants, particularly trees, which would not only amplify the sound of leaves blowing in the wind but also attract a greater variety of birds and insects, thereby enriching the diversity of natural sounds on the school environment.

Based on these observations, the study investigated the different dimensions of single sound sources and their correlation with PRS. The results indicated that most natural sound frequencies, such as birds chirping, leaves blowing in the wind, and running water, were positively correlated with PRS. Therefore, natural sounds with higher frequencies evoke better restorative feelings in students. These findings align with those of previous research, further demonstrating that natural sounds can offer positive restorative experiences and benefit human health (Zhu et al., 2023; Ratcliffe, 2021; Li Z. Z. et al., 2021; Li S. et al., 2021; Li H. et al., 2021; Zhao et al., 2020; Li and Kang, 2019). The frequency and loudness of artificial sounds generated by student movements demonstrated a positive correlation with PRS, such as the sounds generated when playing football, badminton, and table tennis. These sounds were not perceive as noise, but rather improved the students’ perception of restorative soundscape evaluation. The preference for and match of artificial sounds related to students’ learning, such as lecture and reading sounds—which also had high-evaluation indices—showed a positive correlation with PRS, confirming the argument that certain artificial sounds are also associated with restorative benefits. Therefore, “human” sounds derived from human activities are significant (Jo and Jeon, 2020). When designing optimal school environment soundscapes, it is essential to improve the infrastructure for student activities or to provide relevant sounds through hidden speakers. Most mechanical sounds, such as the sound of bells ringing, campus radio, and construction noise, were positively correlated with PRS in terms of preference and matching degree. This findings may be because that both “positive” and “negative” sound sources can be found in the same category of sound and because bells, radio, and construction were considered together, where “positive” sound sources prevailed in this specific study. When the preference value of these mechanical sounds is higher and more integrated with the school environment, students experience better restorative feelings. However, construction noise received the lowest scores for preference and compatibility among various sounds, indicating that its presence diminishes students’ restorative feelings. This finding is consistent with previous studies. By contrast, campus radio and school bell sounds received higher preferences and matching scores, contributing positively to restorative soundscapes. In other words, the matching of sound sources will affect students’ PRS.

### 4.4 Effects of audiovisual interaction on PRS

Soundscapes are intricately linked to the surrounding landscape, and these factors influence each other (Li and Lau, 2020). The results of the study indicated that the combination of sound and vision influenced students’ PRS, with auditory perception having a greater influence on these perceptions. Among the observed variables of auditory perception, the appropriateness of the soundscape had the greatest influence. The study also showed that visual factors played a mediating role in the model of the effect of PRS. In other words, auditory perception has both direct and indirect roles in contributing to the PRS. Auditory perception can directly influence and significantly contribute positively to the PRS. Additionally, it can be complemented by visual landscape perception. This suggests that combined audiovisual stimulation can enhance the PRS more than sensory stimulation alone (Li Z. Z. et al., 2021; Li S. et al., 2021; Li H. et al., 2021; Guo et al., 2022). Therefore, in the process of landscape design, multisensory interactions and coordination must be emphasized to achieve higher restoration.

### 4.5 Analysis of the key factors affecting the PRS

To clarify the factors influencing PRS, a multiple stepwise regression analysis was conducted using the average PRS score as the dependent variable, while sound levels, personal characteristics, sound types, and audiovisual dimensions served as independent variables. The analysis confirmed that the tolerance for independent variables was greater than 0.1 and the variance inflation factor was less than 5, indicating no collinearity among the independent variables. The detailed model is presented in Table 6 (*F* = 29.494, *p* = 0.376). Among all the factors analyzed, soundscape appropriateness had the most significant impact on PRS (*β* = 0.311, *p* < 0.01). This indicates that pleasant, interesting, energetic, comfortable, and favorable soundscapes contribute substantially to mood recovery and stress relief (Guo et al., 2022). The second most significant factor was visual landscape evaluation (*β* = 0.178, *p* < 0.01), suggesting that a school sound environment characterized by interesting, harmonious, and attractive landscapes effectively alleviates fatigue and reduces stress. Notably, mechanical loudness had a significant negative effect (*β* = −0.132, *p* < 0.01), indicating that as mechanical loudness increased, PRS decreased.

**Table 6:** Multiple stepwise regression analysis.

Independent variables	Standardized coefficients	t	Tolerance	VIF	R^2^	F
Appropriateness	0.311	7.224**	0.842	1.187	0.376	48.494
Visual landscape evaluation	0.178	4.108**	0.831	1.203		
Loudness of mechanical sounds	−0.132	−3.197**	0.912	1.096		
Frequency of natural sounds	0.119	2.906**	0.925	1.081		

**p < 0.01.

### 4.6 Limitations and prospects

This study has some limitations despite its capacity to provide a deeper understanding of the secondary school acoustic environments and the factors influencing the PRS. First, the results of the audiovisual interaction in this study only revealed the connection between environmental perception and the PRS. They did not reveal the physiological and psychological dimensions. Second, the physical indices describing the sound environment of the schools are relatively single; only L_Aeq,10min_ and L_10-90,10min_ were used here. Accordingly, more indices and research methods should be adopted in the future, such as describing the relatively low-frequency content of sound—L_Ceq_-L_Aeq_, and characterizing the spectral composition of the acoustic environment—spectral centroid (Log_G_). These indices can describe more accurately the acoustic environments of schools. Finally, during the measurement process, owing to the random search for students, students were not always available to fill out the questionnaire during each measurement time, which was a limitation in the field survey.

The impact of these factors should be investigated further in future studies to establish more suitable mechanisms for influencing PRS for secondary school students. In addition, future studies should explore the actual restorative effects of various sounds on secondary school students, considering their physiological, psychological, attentional, and stress-related aspects.

## 5 Conclusion

This study investigated the factors affecting PRS in terms of four aspects through questionnaires and field measurements, using six secondary schools in the Yangling District as study sites. The findings are summarized as follows:

1. The average L_Aeq,10min_ and L_10-90,10min_ values for the studied school environments were 56.2 and 8.37 dB(A), respectively. When L_Aeq_ was below 59 dB(A), a positive correlation with PRS was observed.
2. Gender, stress level, attention level, and noise disturbance were found to be closely related to PRS.
3. The frequency of natural and artificial sounds generated by student movements showed a positive correlation with PRS. Additionally, the preference for and matching of most mechanical sounds were positively correlated with PRS.
4. Both sound and vision positively contributed to PRS, with sound perception exerting a greater influence and visual factors serving a mediating role.
5. The most critical factor influencing PRS was soundscape appropriateness, followed by visual landscape evaluation and the frequency of natural sounds.

The results indicate that the consistency of audiovisual perception and the type of sound are important indices for designing restorative soundscapes. These findings provide a theoretical basis for optimizing school environment soundscapes.

## Data Availability

The original contributions presented in the study are included in the article/Supplementary material, further inquiries can be directed to the corresponding author/s.

## References

[ref1] Aletta F. Oberman T. Kang J. (2018). Positive health-related effects of perceiving urban soundscapes: a systematic review. Lancet 392:S3. doi: 10.1016/S0140-6736(18)32044-0 PMC6266166 30380601

[ref2] Chen Q. (2021). A study on evaluation and construction of soundscape on college campus. (master’s thesis, Guangxi University).

[ref3] Conservatory of Pavia Carugno G. (2022). Music as a tool to express emotions: remembering the quarantine soundscape at the middle school. Sy 23, 127–138. doi: 10.46522/S.2022.S1.14

[ref4] de Paiva Vianna K. M. Rodrigues R. M. C. Alves Cardoso M. R. (2015). Noise pollution and annoyance: an urban soundscapes study. Noise Health 17, 125–133. doi: 10.4103/1463-1741.155833, PMID: 25913551 PMC4918656

[ref5] Deb S. Strodl E. Sun J. (2015). Academic stress, parental pressure, anxiety and mental health among Indian high school students. Intl J Phys Beh Res. 5, 26–34. doi: 10.5923/j.ijpbs.20150501.04

[ref6] Di H. Liu X. Zhang J. Tong Z. Ji M. Li F.. (2018). Estimation of the quality of an urban acoustic environment based on traffic noise evaluation models. Appl. Acoust. 141, 115–124. doi: 10.1016/j.apacoust.2018.07.010

[ref7] Erfanian M. Mitchell A. Aletta F. Kang J. (2021). Psychological well-being and demographic factors can mediate soundscape pleasantness and eventfulness: a large sample study. J. Environ. Psychol. 77:101660. doi: 10.1016/j.jenvp.2021.101660

[ref8] Ganic E. Babic O. Cangalovic M. Stanojevic M. (2018). Air traffic assignment to reduce population noise exposure using activity-based approach. Transport. Res. Part D Transport Environ. 63, 58–71. doi: 10.1016/j.trd.2018.04.012

[ref9] Gao Jianmin L. Y. Shihe T. Yuqiao Q. (2023). Assessment of acoustic comfort in twin pagoda Temple, Taiyuan, and its influencing factors. Shanxi Architect. 49, 32–38. doi: 10.13719/j.cnki.1009-6825.2023.11.009

[ref10] Grahn P. Stigsdotter U. K. (2010). The relation between perceived sensory dimensions of urban green space and stress restoration. Landsc. Urban Plan. 94, 264–275. doi: 10.1016/j.landurbplan.2009.10.012

[ref11] Guo Y. L. Jiang X. M. Zhang L. F. Zhang H. Jiang Z. Q. (2022). Effects of sound source landscape in urban Forest Park on alleviating mental stress of visitors: Evidence from Huolu Mountain Forest Park, Guangzhou. Sustainability 14:14. doi: 10.3390/su142215125

[ref12] Guo X. Liu J. Albert C. Hong X. C. (2022). Audio-visual interaction and visitor characteristics affect perceived soundscape restorativeness: case study in five parks in China. Urban For. Urban Green. 77:127738. doi: 10.1016/j.ufug.2022.127738

[ref13] Herranz-Pascual K. Aspuru I. Iraurgi I. Santander A. Eguiguren J. L. García I. (2019). Going beyond quietness: determining the emotionally restorative effect of acoustic environments in urban open public spaces. Int. J. Environ. Res. Public Health 16:1284. doi: 10.3390/ijerph16071284, PMID: 30974811 PMC6479382

[ref14] Hong J. Y. Jeon J. Y. (2013). Designing sound and visual components for enhancement of urban soundscapes. J. Acoust. Soc. Am. 134, 2026–2036. doi: 10.1121/1.4817924, PMID: 23967935

[ref15] Huang X. F. Liu J. Y. Meng Z. L. (2022). Application of university campus noise map based on noise propagation model: a case in Guangxi university. Sustain. For. 14:8613. doi: 10.3390/su14148613

[ref16] Jeon J. Y. Hwang I. H. Hong J. Y. (2014). Soundscape evaluation in a Catholic cathedral and Buddhist temple precincts through social surveys and soundwalks. J. Acoust. Soc. Am. 135, 1863–1874. doi: 10.1121/1.4866239, PMID: 25234985

[ref17] Ji Xianrong L. F. Yaping W. (2017). Acoustic comfort in university campuses’ outdoor learning spaces—based on 5 university libraries’ outdoor space in Shanxi. J. Appl. Acoust. 36, 311–316. doi: 10.11684/j.issn.1000-310X.2017.04.005

[ref18] Jing Y. Hongda W. Junming Z. Yueyan L. Lingyan C. Yushan Z. (2022). Difference in soundscape experience of historic blocks based on different soundscape preferences. J. Chin. Urban Forestry 20, 55–60. doi: 10.12169/zgcsly.2021.09.07.0002

[ref19] Jo H. I. Jeon J. Y. (2020). Effect of the appropriateness of sound environment on urban soundscape assessment. Build. Environ. 179:106975. doi: 10.1016/j.buildenv.2020.106975

[ref20] Kang J. Zhang M. (2010). Semantic differential analysis of the soundscape in urban open public spaces. Build. Environ. 45, 150–157. doi: 10.1016/j.buildenv.2009.05.014

[ref21] Kelly S. M. Walton H. R. (2021). "I'll work out tomorrow": the procrastination in exercise scale. J. Health Psychol. 26, 2613–2625. doi: 10.1177/1359105320916541, PMID: 32459106

[ref22] Kraus N. Slater J. Thompson E. C. Hornickel J. Strait D. L. Nicol T.. (2014). Auditory learning through active engagement with sound: biological impact of community music lessons in at-risk children. Front. Neurosci. 8:351. doi: 10.3389/fnins.2014.00351 25414631 PMC4220673

[ref23] Krzywicka P. Byrka K. (2017). Restorative qualities of and preference for natural and urban soundscapes. Front. Psychol. 8:01705. doi: 10.3389/fpsyg.2017.01705, PMID: 29046653 PMC5632731

[ref24] Li Z. Z. Ba M. H. Kang J. (2021). Physiological indicators and subjective restorativeness with audio-visual interactions in urban soundscapes. Sustain. Cities Soc. 75:103360. doi: 10.1016/j.scs.2021.103360

[ref25] Li S. Ding Q. Yuan Y. C. Yue Z. Z. (2021). Audio-visual causality and stimulus reliability affect audio-visual synchrony perception. Front. Psychol. 12:629996. doi: 10.3389/fpsyg.2021.629996 33679553 PMC7930005

[ref26] Li Z. Z. Kang J. (2019). Sensitivity analysis of changes in human physiological indicators observed in soundscapes. Landsc. Urban Plan. 190:103593. doi: 10.1016/j.landurbplan.2019.103593

[ref27] Li H. Lau S. K. (2020). A review of audio-visual interaction on soundscape assessment in urban built environments. Appl. Acoust. 166:107372. doi: 10.1016/j.apacoust.2020.107372

[ref28] Li H. Xie H. Woodward G. (2021). Soundscape components, perceptions, and EEG reactions in typical mountainous urban parks. Urban For. Urban Green. 64:127269. doi: 10.1016/j.ufug.2021.127269

[ref29] Liu Jiang G. X. Xinchen H. Xuewei Z. (2022). The impact of individual factors on perceived soundscape Restorativeness in urban parks. Chin. Landscape Architect. 38, 40–45. doi: 10.19775/j.cla.2022.09.0040

[ref30] Lu X. D. Tang J. Zhu P. S. Guo F. Cai J. Zhang H. C. (2020). Spatial variations in pedestrian soundscape evaluation of traffic noise. Environ. Impact Assess. Rev. 83:106399. doi: 10.1016/j.eiar.2020.106399

[ref31] Luo L. Zhang Q. Mao Y. Peng Y. Wang T. Xu J. (2023). A study on the soundscape preferences of the elderly in the urban Forest parks of underdeveloped cities in China. Forests 14:1266. doi: 10.3390/f14061266

[ref32] Ma K. W. Mak C. M. Wong H. M. (2021). Effects of environmental sound quality on soundscape preference in a public urban space. Appl. Acoust. 171:107570. doi: 10.1016/j.apacoust.2020.107570

[ref33] Ma K. W. Wong H. M. Mak C. M. (2018). A systematic review of human perceptual dimensions of sound: Meta-analysis of semantic differential method applications to indoor and outdoor sounds. Build. Environ. 133, 123–150. doi: 10.1016/j.buildenv.2018.02.021

[ref34] Payne S. R. (2008). Are perceived soundscapes within urban parks restorative. J. Acoust. Soc. Am. 123:3809. doi: 10.1121/1.2935525

[ref35] Petrovici A. M. Cueto J. L. Gey R. Nedeff F. Hernandez R. Tomozei C.. (2016). Optimization of some alternatives to noise barriers as noise mitigation measures on major roads in EUROPE. Case study of a highway in SPAIN. Environ. Eng. Manag. J. 15, 1617–1628. doi: 10.30638/eemj.2016.174

[ref36] Preacher K. J. Hayes A. F. (2008). Asymptotic and resampling strategies for assessing and comparing indirect effects in multiple mediator models. Behav. Res. Methods 40, 879–891. doi: 10.3758/BRM.40.3.879, PMID: 18697684

[ref37] Qiu Z. H. Guo Y. Wang J. Zhang H. B. (2022). Associations of parenting style and resilience with depression and anxiety symptoms in Chinese middle school students. Front. Psychol. 13:897339. doi: 10.3389/fpsyg.2022.897339, PMID: 35846635 PMC9285101

[ref38] Ratcliffe E. (2021). Sound and soundscape in restorative natural environments: a narrative literature review. Front. Psychol. 12:570563. doi: 10.3389/fpsyg.2021.570563, PMID: 33981262 PMC8107214

[ref39] Shao Y. Hao Y. Yin Y. Yu M. Xue Z. (2022). Improving soundscape comfort in urban green spaces based on aural-visual interaction attributes of landscape experience. Forests 13:1262. doi: 10.3390/f13081262

[ref40] Shu S. (2023). Exploring the role of soundscape in restorative experience: a pilot study from children's perspective. Front. Psychol. 14:1131170. doi: 10.3389/fpsyg.2023.1131170, PMID: 36998354 PMC10043254

[ref41] Shu S. Ma H. (2020). Restorative effects of urban park soundscapes on children's psychophysiological stress. Appl. Acoust. 164:107293. doi: 10.1016/j.apacoust.2020.107293

[ref42] Sliwinska-Kowalska M. Zaborowski K. (2017). WHO environmental noise guidelines for the European region: a systematic review on environmental noise and permanent hearing loss and tinnitus. Int. J. Environ. Res. Public Health 14:1139. doi: 10.3390/ijerph14101139, PMID: 28953238 PMC5664640

[ref43] Smalley A. J. White M. P. Sandiford R. Desai N. Watson C. Smalley N.. (2023). Soundscapes, music, and memories: exploring the factors that influence emotional responses to virtual nature content. J. Environ. Psychol. 89:102060. doi: 10.1016/j.jenvp.2023.102060

[ref44] Steele D. Fraisse V. Bild E. Guastavino C. (2021). Bringing music to the park: the effect of Musikiosk on the quality of public experience. Appl. Acoust. 177:107910. doi: 10.1016/j.apacoust.2021.107910

[ref45] Szkopiecka A. Wyrwa J. P. Chrobak G. Kolodynska I. Szewranski S. (2023). Perceived restorative potential of urban parks by citizens—a case study from Wrocław, Poland. Sustainability 15:7912. doi: 10.3390/su15107912

[ref46] Weinstein (1978). Individual differences in reactions to noise: a longitudinal study in a college dormitory. J. Appl. Psychol. 63, 458–466. doi: 10.1037/0021-9010.63.4.458, PMID: 701213

[ref47] Wen X. Lin Y. Liu Y. Starcevich K. Yuan F. Wang X.. (2020). A latent profile analysis of anxiety among junior high school students in less developed rural regions of China. Int. J. Environ. Res. Public Health 17:4079. doi: 10.3390/ijerph17114079, PMID: 32521646 PMC7312008

[ref48] Yufeng L. Xiaofeng S. (2020). Investigation and evaluation of acoustical environment in Pingyao Ancient City. Tech. Acoust. 39, 728–735. doi: 10.16300/j.cnki.1000-3630.2020.06.013

[ref49] Zhang D. X. Zhang M. Liu D. P. Kang J. (2016). Soundscape evaluation in Han Chinese Buddhist temples. Appl. Acoust. 111, 188–197. doi: 10.1016/j.apacoust.2016.04.020

[ref50] Zhao W. Li H. Zhu X. Ge T. (2020). Effect of birdsong soundscape on perceived Restorativeness in an Urban Park. Int. J. Environ. Res. Public Health 17:5659. doi: 10.3390/ijerph17165659, PMID: 32764453 PMC7459586

[ref51] Zhao J. W. Xu W. Y. Ye L. (2018). Effects of auditory-visual combinations on perceived restorative potential of urban green space. Appl. Acoust. 141, 169–177. doi: 10.1016/j.apacoust.2018.07.001

[ref52] Zhu Y. Huang N. Weng Y. Tong H. Wang X. Chen J.. (2023). Does soundscape perception affect health benefits, as mediated by restorative perception? Forests 14:1798. doi: 10.3390/f14091798

