# Supplementary material for: Influence of sound levels, secondary school student characteristics, sound types, and audiovisual interactions on the restorative potential of school environment soundscapes
Source: Front Psychol. 2025 Feb 12;15:1476553. doi: 10.3389/fpsyg.2024.1476553 (PMC11864134; doi:10.3389/fpsyg.2024.1476553)
Supplement: Supplementary file 1 [file Table_1.DOCX]

Appendix A

TableA.1 Basic Information

| Category | options |
| --- | --- |
| Sex | 🞎Boy 🞎Girl |
| Age | 🞎12 🞎13 🞎14 🞎15 🞎16 🞎17 🞎18 |
| Grade | 🞎First year of junior high 🞎Second year of junior high  🞎Third year of junior high 🞎First year of senior high  🞎Second year of senior high 🞎Third year of senior high |
| School | 🞎School A 🞎School B 🞎School C  🞎School D 🞎School E 🞎School F |

TableA.2 the state of secondary school students

| Measurement indicators | Specific issues | Evaluation methods |
| --- | --- | --- |
| Stress Level | Over the past month, have you found it difficult to calm yourself down? | Four-point Likert scale |
|  | Over the past month, have you tended to react excessively to things? |  |
|  | Over the past month, have you felt like you've expended a lot of energy? |  |
|  | Over the past month, have you felt restless and uneasy? |  |
|  | Over the past month, have you found it difficult to relax? |  |
|  | Over the past month, have you been unable to tolerate anything that hinders your ability to continue studying or living? |  |
|  | Over the past month, have you noticed that you are easily angered? |  |
| Attention Level | Do you feel tired and have a decrease in attention while studying and living? | Five-point Likert scale |
| Restorative Needs | Do you feel the need to find ways to release stress recently? |  |
|  | Do you feel the need to find ways to relieve fatigue recently? |  |
| Noise Sensitivity | In the past month, have you felt nervous and agitated due to noise at times? |  |
|  | In the past month, have you been able to concentrate well regardless of what is happening around you? |  |
|  | In the past month, have you been sensitive to noise? |  |
| Level of Disturbance by Noise | In the past month, have you been disturbed by noise? |  |
|  | In the past month, has noise consumed a lot of your energy? |  |
|  | In the past month, has noise affected your studying or daily life? |  |
| Sources of stress | 🞎Interpersonal relationship 🞎Academic aspect 🞎Family relationship  🞎Future prospects 🞎Economic aspect 🞎Health aspect  🞎Teacher-student relationship | Multiple choice |
| A way to relieve fatigue and stress | 🞎Ask teachers and friends for help 🞎Let off steam  🞎Self-analysis 🞎problem solving  🞎Divert attention by playing sports on campus🞎Silent patience  🞎Escape the problem 🞎fantasy |  |

TableA.3 Evaluation of audiovisual environment

| Measurement indicators | Specific issues | Evaluation methods |
| --- | --- | --- |
| Visual assessment | comfortable-unpleasant | Five-point bipolar rating scale |
|  | open-closed |  |
|  | interesting-uninteresting |  |
|  | harmonious-chaotic |  |
|  | attractive-uninteresting |  |
| Auditory assessment | artificial-natural |  |
|  | likeable-disgusted |  |
|  | interesting-boring |  |
|  | pleasant-sad |  |
|  | vibrant–listless |  |
|  | comfortable-uncomfortable |  |
|  | concentrated-dispersed |  |
|  | coordinated-disorganized |  |
|  | rich-simple |  |
|  | varied–monotonous |  |
|  | quiet-noisy |  |
|  | friendly-hostile |  |
|  | harmonious–chaotic |  |
|  | safe-dangerous |  |
|  | weak-strong |  |

TableA.4 Evaluation of the sound

| Measurement indicators | Specific issues | Evaluation methods |
| --- | --- | --- |
| Frequency, loudness, preference and Match | The sound of talking | Five-point Likert scale |
|  | Class bells |  |
|  | Playing basketball |  |
|  | The wind |  |
|  | Footsteps |  |
|  | Birds chirping |  |
|  | the sound of campus announcements |  |
|  | Reading |  |
|  | Lecture |  |
|  | Playing soccer |  |
|  | Whistle |  |
|  | Playing table tennis |  |
|  | Leaves blowing in the wind |  |
|  | Motorized vehicles |  |
|  | Badminton |  |
|  | Rain |  |
|  | Insects chirping |  |
|  | Running water |  |
|  | Construction noise |  |

TableA.5 the Perceived Restorativeness Soundscape Scale

| Measurement indicators | Specific issues | Evaluation methods |
| --- | --- | --- |
| Fascination | I find the sounds on campus very appealing. | Five-point Likert scale |
|  | My attention is drawn to many interesting sounds on campus. |  |
|  | The sounds on campus make me want to linger here. |  |
|  | The sounds on campus make me curious about things on campus. |  |
|  | The sound environment on campus captivates my full attention. |  |
| Being-away-to | The sounds on campus are different from what I have heard before. |  |
|  | The sound environment on campus is different from what I hear in my daily life. |  |
| Being-away-from | The sound environment on campus helps me avoid unnecessary distractions. |  |
|  | The sounds on campus allow me to relax from my daily academic life. |  |
|  | The sounds on campus free me from the sounds I hear in my daily life. |  |
| Compatibility | The sounds on campus are related to some activities I enjoy doing. |  |
|  | The sound environment on campus aligns well with my personal preferences. |  |
| Coherence | All the sounds I hear on campus match the campus environment. |  |
|  | All the sounds blend together to form a harmonious sound environment. |  |
|  | The sounds I hear seem to naturally blend with the campus. |  |
