# Supplementary material for: Influence of sound levels, secondary school student characteristics, sound types, and audiovisual interactions on the restorative potential of school environment soundscapes
Source: Front Psychol. 2025 Feb 12;15:1476553. doi: 10.3389/fpsyg.2024.1476553 (PMC11864134; doi:10.3389/fpsyg.2024.1476553)
Supplement: Supplementary file 2 [file Table_2.DOCX]

Appendix B

Table B.1 Sound pressure level measurement and questionnaire distribution details

| School | Indicators | P1 | P2 | P3 | P4 | P5 | P6 |
| --- | --- | --- | --- | --- | --- | --- | --- |
| School A | LAeq/dB | 58.20 | 58.50 | 55.90 | 58.50 | 58.50 | 50.60 |
|  | L10-90/dB | 8.20 | 11.29 | 7.38 | 8.21 | 7.53 | 6.42 |
|  | Number of questionnaires | 14 | 12 | 14 | 15 | 13 | 15 |
| School B | LAeq/dB | 57.01 | 59.77 | 56.19 | 53.43 | 54.69 | 58.33 |
|  | L10-90/dB | 8.92 | 6.32 | 5.38 | 5.98 | 8.12 | 8.68 |
|  | Number of questionnaires | 15 | 14 | 15 | 15 | 13 | 16 |
| School C | LAeq/dB | 58.06 | 60.06 | 51.70 | 57.51 | 52.56 | 51.93 |
|  | L10-90/dB | 10.65 | 6.51 | 5.50 | 8.91 | 8.02 | 9.47 |
|  | Number of questionnaires | 12 | 13 | 15 | 13 | 14 | 13 |
| School D | LAeq/dB | 59.89 | 57.86 | 52.07 | 53.73 | 53.11 | 52.00 |
|  | L10-90/dB | 6.13 | 5.47 | 5.23 | 6.58 | 7.23 | 5.45 |
|  | Number of questionnaires | 13 | 12 | 14 | 13 | 13 | 12 |
| School E | LAeq/dB | 59.67 | 59.93 | 59.79 | 56.01 | 55.67 | 58.20 |
|  | L10-90/dB | 10.87 | 12.47 | 10.58 | 10.78 | 8.93 | 10.75 |
|  | Number of questionnaires | 13 | 12 | 12 | 12 | 13 | 13 |
| School F | LAeq/dB | 55.37 | 58.73 | 55.57 | 55.73 | 54.80 | 53.97 |
|  | L10-90/dB | 8.88 | 9.13 | 9.85 | 11.50 | 10.03 | 12.25 |
|  | Number of questionnaires | 13 | 12 | 13 | 14 | 14 | 13 |

Table B.2 Validation of the reliability and validity of the scale

| Category | Cronbach's alpha | KMO | Number of items |
| --- | --- | --- | --- |
| Spatial evaluation of landscape features | 0.710 | 0.847 | 2 |
| Visual Landscape Evaluation | 0.791 |  | 3 |
| **Visual perception** | **0.849** |  | **5** |
| Appropriateness | 0.847 | 0.908 | 5 |
| Stability | 0.747 |  | 3 |
| Native | 0.703 |  | 3 |
| Richness | 0.672 |  | 2 |
| Harmony | 0.702 |  | 2 |
| **Auditory perception** | **0.893** |  | **15** |
| Fascination | 0.857 | 0.887 | 5 |
| Being-away-to | 0.654 |  | 2 |
| Being-away-from | 0.748 |  | 3 |
| Compatibility | 0.662 |  | 2 |
| Coherence | 0.780 |  | 3 |
| **Soundscape restorative perception** | **0.879** |  | **15** |
| Stress level | 0.775 | 0.748 | 7 |
| Noise sensitivity | 0.789 | 0.763 | 3 |
| Degree of disturbance by noise | 0.791 | 0.697 | 3 |
| Need for restoration | 0.831 | 0.768 | 2 |
