# Supplementary material for: Influence of sound levels, secondary school student characteristics, sound types, and audiovisual interactions on the restorative potential of school environment soundscapes
Source: Front Psychol. 2025 Feb 12;15:1476553. doi: 10.3389/fpsyg.2024.1476553 (PMC11864134; doi:10.3389/fpsyg.2024.1476553)
Supplement: Supplementary file 3 [file Table_3.DOCX]

Appendix C

TableC.1 Factor loadings of the principal component analysis in perceptions of visual environments

| Group | Attributes | Component 1 | Component 2 |
| --- | --- | --- | --- |
| Landscape Character Spatial Assessment | Comfortable | 0.715 |  |
|  | Open | 0.888 |  |
| Visual Landscape Evaluation | Interesting |  | 0.751 |
|  | Harmonious |  | 0.871 |
|  | Attractive |  | 0.638 |

TableC.2 Factor loadings of the principal component analysis in perceptions of Auditory environments

| Group | Attributes | Component 1 | Component 2 | Component 3 | Component 4 | Component 5 |
| --- | --- | --- | --- | --- | --- | --- |
| Appropriateness | Pleasant | 0.784 |  |  |  |  |
|  | Interesting | 0.765 |  |  |  |  |
|  | Energetic | 0.732 |  |  |  |  |
|  | Comfortable | 0.610 |  |  |  |  |
|  | Favorable | 0.566 |  |  |  |  |
| Stability | Harmonious |  | 0.821 |  |  |  |
|  | Safe |  | 0.734 |  |  |  |
|  | Friendly |  | 0.674 |  |  |  |
| Native | Natural |  |  | 0.805 |  |  |
|  | Quiet |  |  | 0.768 |  |  |
|  | Weak |  |  | 0.639 |  |  |
| Abundance | Rich |  |  |  | 0.782 |  |
|  | Diverse |  |  |  | 0.780 |  |
| Harmonious | Concentrated |  |  |  |  | 0.841 |
|  | Harmonized |  |  |  |  | 0716 |
