# Supplementary material for: Influence of sound levels, secondary school student characteristics, sound types, and audiovisual interactions on the restorative potential of school environment soundscapes
Source: Front Psychol. 2025 Feb 12;15:1476553. doi: 10.3389/fpsyg.2024.1476553 (PMC11864134; doi:10.3389/fpsyg.2024.1476553)
Supplement: Supplementary file 4 [file Table_4.DOCX]

Appendix D

TableD.1 Convergent Validity and Combined Reliability Tests for Various Dimensions of the Scale

| Latent variables | Observed variables | Estimate | AVE | CR |
| --- | --- | --- | --- | --- |
| Auditory perception | Interesting <--- Appropriateness | 0.678*** | 0.522 | 0.845 |
|  | Pleasant <--Appropriateness | 0.767*** |  |  |
|  | Vibrant <--- Appropriateness | 0.731*** |  |  |
|  | Favorable <--- Appropriateness | 0.690*** |  |  |
|  | Comfortable <--- Appropriateness | 0.743*** |  |  |
|  | Friendly <--- Stability | 0.809*** | 0.543 | 0.780 |
|  | Harmonious <-- Stability | 0.688*** |  |  |
|  | Safe <-- Stability | 0.708*** |  |  |
|  | Quiet <--- Native | 0.656*** | 0.527 | 0.769 |
|  | Weak <--- Native | 0.805*** |  |  |
|  | Natural <--- Native | 0.710*** |  |  |
|  | Rich <--- Abundance | 0.655*** | 0.560 | 0.715 |
|  | Diverse <--- Abundance | 0.831*** |  |  |
|  | Harmonized <-- Harmonious | 0.743*** | 0.508 | 0.674 |
|  | Concentrated <-- Harmonious | 0.682*** |  |  |
| Visual perception | Comfortable <--- Landscape Character Spatial Assessment | 0.792*** | 0.555 | 0.713 |
|  | Open <--- Spatial evaluation of landscape features | 0.695*** |  |  |
|  | Interesting <--- Visual Landscape Evaluation | 0.789*** | 0.559 | 0.797 |
|  | Harmonious <--- Visual Landscape Evaluation | 0.649*** |  |  |
|  | Attractive <--- Visual Landscape Evaluation | 0.814*** |  |  |
| Soundscape restorative perception | Q1<---Fascination | 0.744*** | 0.560 | 0.863 |
|  | Q2<---Fascination | 0.707*** |  |  |
|  | Q3<---Fascination | 0.817*** |  |  |
|  | Q4<---Fascination | 0.752*** |  |  |
|  | Q5<---Fascination | 0.713*** |  |  |
|  | Q6<---Being-away-to | 0.842*** | 0.626 | 0.761 |
|  | Q7<--- Being-away-to | 0.723*** |  |  |
|  | Q8<---Being-away-from | 0.562*** | 0.586 | 0.804 |
|  | Q9<---Being-away-from | 0.849*** |  |  |
|  | Q10<---Being-away-from | 0.849*** |  |  |
|  | Q11<---Compatibility | 0.785*** | 0.601 | 0.751 |
|  | Q12<---Compatibility | 0.766*** |  |  |
|  | Q13<---Coherence | 0.700*** | 0.580 | 0.805 |
|  | Q14<---Coherence | 0.796*** |  |  |
|  | Q15<---Coherence | 0.786*** |  |  |

Note:*p<0.05,**p<0.01,***p<0.001

TableD.2 Distinction validity test for each dimension of auditory perception

| Variable | Appropriateness | Stability | Native | Richness | Harmony |
| --- | --- | --- | --- | --- | --- |
| Appropriateness | **0.522** |  |  |  |  |
| Stability | 0.668 | **0.543** |  |  |  |
| Native | 0.641 | 0.502 | **0.527** |  |  |
| Richness | 0.767 | 0.533 | 0.558 | **0.560** |  |
| Harmony | 0.725 | 0.643 | 0.414 | 0.629 | **0.508** |
| Square root of AVE value | 0.722 | 0.737 | 0.726 | 0.748 | 0.713 |

TableD.3 Distinction validity test for each dimension of visual perception

| Variable | Landscape Character Spatial Assessment | Visual Landscape Evaluation |
| --- | --- | --- |
| Landscape Character Spatial Assessment | **0.555** |  |
| Visual Landscape Evaluation | 0.714 | **0.559** |
| Square root of AVE value | 0.745 | 0.748 |

TableD.4 Distinction validity test for each dimension of soundscape restorative perception

| Variable | Fascination | Being-away-to | Being-away-from | Compatibility | Coherence |
| --- | --- | --- | --- | --- | --- |
| Fascination | **0.560** |  |  |  |  |
| Being-away-to | 0.419 | **0.626** |  |  |  |
| Being-away-from | 0.662 | 0.406 | **0.586** |  |  |
| Compatibility | 0.648 | 0.339 | 0.696 | **0.601** |  |
| Coherence | 0.501 | 0.308 | 0.478 | 0.559 | **0.580** |
| Square root of AVE value | 0.748 | 0.791 | 0.766 | 0.775 | 0.762 |
